# Meat in a Seat: A Grounded Theory Study Exploring Moral Injury in Canadian Public Safety Communicators, Firefighters, and Paramedics

**DOI:** 10.3390/ijerph182212145

**Published:** 2021-11-19

**Authors:** Lorraine Smith-MacDonald, Liana Lentz, David Malloy, Suzette Brémault-Phillips, R. Nicholas Carleton

**Affiliations:** 1 Heroes in Mind, Advocacy and Research Consortium, Faculty of Rehabilitation Medicine, University of Alberta, Edmonton, AB T6G 2G4, Canada; llentz@ualberta.ca (L.L.); suzette2@ualberta.ca (S.B.-P.); 2 Faculty of Health Sciences, Western University, London, ON N6A 2M3, Canada; 3 Faculty of Science, Thompsons River University, Kamloops, BC V2C 0C8, Canada; 4 King’s University College, Western University, London, ON N6A 2M3, Canada; david.malloy@kings.uwo.ca; 5 Department of Psychology, University of Regina, Regina, SK S4S 0A2, Canada; nick.carleton@uregina.ca

**Keywords:** moral injury, public safety personnel, paramedics, firefighters, dispatchers, communicators, mental health, trauma

## Abstract

The work of public safety personnel (PSP) is inherently moral; however, the ability of PSP to do what is good and right can be impeded and frustrated, leading to moral suffering. Left unresolved, moral suffering may develop into moral injury (MI) and potential psychological harm. The current study was designed to examine if MI is relevant to frontline public safety communicators, firefighters, and paramedics. Semi-structured interviews (*n* = 3) and focus groups (*n* = 3) were conducted with 19 participants (public safety communicators (*n* = 2); paramedics (*n* = 7); and firefighters (*n =* 10)). Interviews and focus groups were audio-recorded, transcribed, coded, and constantly compared in accordance with the grounded theory method. A conceptual theory of *“frustrating moral expectations”* emerged, with participants identifying three interrelated properties as being potentially morally injurious: chronic societal problems, impaired systems, and organizational quagmires. Participants navigated their moral frustrations through both integrative and disintegrative pathways, resulting in either needing to escape their moral suffering or transforming ontologically. The current study results support MI as a relevant concept for frontline PSP. Given the seriousness of PSP leaving their profession or committing suicide to escape moral suffering, the importance of the impact of MI on PSP and public safety organizations cannot be ignored or underestimated. Understanding the similarities and differences of morally injurious exposures of frontline PSP may be critical for determining mental health and resilience strategies that effectively protect PSP.

## 1. Introduction

Public safety personnel (PSP) are frequently confronted with PMIEs, and often have to make decisions where there is no “right” answer or where they must act against the interests of someone [1,2]. Most people who choose to serve as PSP, including but not limited to, border services officers, public safety communicators (i.e., call taker/dispatchers), correctional workers, firefighters (career and volunteer), Indigenous emergency managers, operational intelligence personnel, paramedics, police (municipal, provincial, federal), and search and rescue personnel [3], do so with a desire to help [4,5,6]. Accordingly, when unable to help or unable to act according to their personal values, PSP may experience moral suffering. This moral suffering, if left unresolved, may then develop into a trauma syndrome called moral injury (MI) [7].

### 1.1. Morals and Moral Injury

Morals, emerging from universal laws of human nature (e.g., the preservation of life or the species, seeking truth, law, and order), set the foundation for how societies, organizations, and individuals behave, regardless of personal characteristics such as ethnicity, gender, and age [8,9]. Morals have been proposed as what differentiates humans from other sentient beings and outline societal beliefs of what is good as expressed through values and behaviors [8,9]. Personal beliefs regarding what is right and wrong may be challenged upon exposure to potentially morally injurious events (PMIEs), cause moral suffering, and produce mental health challenges and moral injury. MI is a multi-facetted construct identified during the mental rehabilitation of Vietnam veterans who were observed experiencing deeply embedded moral wounds that changed their sense of self or their being [10,11]. While an understanding of MI is still evolving, common among definitions is some level of moral suffering or a challenge to personal beliefs regarding what is right and wrong [12]. Similar to an individual’s reaction to many potentially psychologically traumatic events [3], individual reactions to PMIEs are based on context; similarly, decision-making during the PMIE may be impacted by contextual elements such as empathy, role responsibility, and expectations [13]. Exposure to PMIEs may lead to cognitive, emotional, existential, or spiritual struggles [14] rather than the fear-based manifestations associated with exposure to potentially psychologically traumatic events [15]. Research on PMIEs and MI is prolific in military contexts [16] and is increasing in healthcare contexts [17]. The available research suggests that exposure to PMIEs, and the experience of MI, are often comorbid with posttraumatic stress disorder (PTSD) [18] and other posttraumatic stress injuries (PTSI), such as major depressive disorder, generalized anxiety disorder, suicidality, and occupational impairment such as burnout, compassion fatigue, increased absenteeism, and leaving the profession early [19,20]. Extrapolating from this military and healthcare literature, it seems reasonable to presume that MI (in some configuration) would be relevant to other trauma-affected populations such as PSP.

### 1.2. Moral Injury in PSP Populations

To date, there is very little research examining MI in PSP, and the available research has been primarily theoretical in nature or has examined constructs related to moral suffering. A recent scoping review of the evidence-based literature did not identify any studies related to MI and PSP; however, it was determined that there was evidence for four MI associated constructs—values, moral decision-making, organizational betrayal, and spirituality—within PSP literature [21].

Police have been a primary PSP group of focus in theoretical research on ethics and behavior. Police officers have discretion when serving and likely try to act in ways that are consistent with their own morals, ethics, values, and beliefs. The dynamic, exigent, uncertain, and threatening environments in which they serve, and in which decisions are often ambiguous at best, may inherently facilitate exposure to PMIEs, potentially resulting moral suffering that can lead to MI. Police officers are also regularly exposed to potentially psychologically traumatic events [3,22] that can intensify moral suffering as a function of making life and death decisions [20]. Exposure to such events is not limited to police officers but transcends across all PSP groups. The different roles and responsibilities between PSP groups, however, can influence their experiences and outcomes of PMIE exposure [13]. The limited research on paramedics suggests they too may be at high risk for MI or moral distress (as understood in healthcare literature). In particular, the imperfect, fluid, and dynamic prehospital environment in which paramedics are required to make complex ethically and morally ambiguous healthcare decisions may be ripe for PMIEs [23,24,25,26]. PMIEs common to paramedics may include feeling disrespected, operating in a negative ethical climate, and acting against a patient’s best interest. One piece of grey literature has also noted an association between MI and suicidal thoughts and behavior [27]. No literature could be found regarding MI and firefighters.

The current study aims to begin to fill the gap in empirical literature and clarify a very abstract and theoretical understanding of MI by: (1) examining if MI is seen as relevant to the experiences of frontline public safety communicators, firefighters, and paramedics; and (2) identifying PMIEs among a diverse sample of PSP. The information gained will be used to further knowledge of MI and PMIEs in PSP, and to inform psychoeducation related to MI for public safety communicators, firefighters, and paramedics.

## 2. Materials and Methods

The current study was conducted using classical grounded theory (GT), an inductive qualitative research method that focuses on understanding social processes and interactions within a specific population [28,29]. Central to a GT study is generating a core concept that can broadly explain what participants consider to be problematic or of central concern. Classical GT is best suited when minimal research exists on a topic or when a particular aspect of a phenomenon is poorly understood. Classical GT was purposefully selected because the conceptual understanding of MI in PSP is extremely limited and current understandings have broadly drawn from military MI literature. The study design allowed the researchers to explore the data with no preconceived notions regarding what or how MI should be conceptualized, or even if MI would be meaningful or applicable among participating PSP.

Potential participants were recruited from two large metropolitan cities in Western Canada. Recruitment posters were sent to PSP organizations (i.e., firefighters, paramedics, public safety communicators, police) with a request that they be circulated to frontline staff. Potential participants were then instructed to contact the researchers directly to learn more about the study and do a small screening interview with a member of the research team. Inclusion criteria were as follows: the individual could speak English; were employed as frontline public safety communicators, firefighters, paramedics, or police; and had at least one year of PSP experience. To ensure lower risk of psychological harm to participants, PSP working in a modified capacity or currently being treated for a mental health condition were excluded. There were 19 participants included in the current study. Confidentiality rules were reviewed, and consent obtained prior to participation. Research Ethics Board (i.e., University of Alberta Pro #00102000, University of Regina REB# 2020-115, Western University Project ID#115902) and PSP organizational approvals were obtained prior to data collection.

Consent forms and demographic questionnaires (e.g., age, sex, gender, profession, organizational affiliation, and years of service) were completed by participants online via REDCap, which is a secure, web-based software platform designed to support data capture for research studies. Participants included 2 public safety communicators (2 males; mean age 35.0 years), 7 paramedics (4 females, 3 males; mean age 37.6 years), and 10 firefighters (10 males; mean age 43.3). The recruitment of police officers during the study period was unsuccessful.

Qualitative data was then collected through semi-structured interviews (*n* = 3) and focus groups (*n* = 3). These interviews were conducted and recorded over encrypted Zoom video conferencing software. Interviews lasted approximately 50–60 min, while focus groups were approximately 90–120 min in length. The research team developed a semi-structured interview guide; however, in accordance with classical GT [28], the interview guide questions were continually refined and focused as the data was collected and analyzed. Initial questions were open-ended and designed to explore participant understandings of MI, while subsequent questions focused on data related to emerging categories of interest to the participants. Data collection occurred concurrently through an iterative process in GT. Data from each interview and focus group were recorded, transcribed, coded, and analyzed before any subsequent interview or focus group session occurred. The collection and analysis process allowed the research team to be sensitive to emerging concepts, refine interview questions, and conduct interviews or focus groups in a more focused manner as key concepts emerged.

The distinguishing feature of GT data analysis is the constant comparative method and the creation of a social theory explaining the participants’ problematic behavior [29]. To develop this theory, data analysis aims to facilitate the emergence of conceptual and core categories, subsequent properties of that category, and a comprehensive explanatory theory. Within GT, a conceptual category is the encompassing explanation, while the properties of the categories are usually associated with particular behaviors and actions [29]. Once the categories are established, one core category is selected which provides a systematic framework upon which the new theory rests. In order to develop these properties and categories, GT requires two overall levels of coding: substantive and theoretical. Following best practices, line-by-line open coding was conducted by hand to compare events that could be conceptualized as PMIEs within and between interviews. In vivo codes were used to maintain authenticity to the language of the participants to avoid imparting biases onto the data. Data were then grouped into categories and properties.

To conduct the theoretical coding the lead researcher began writing memos regarding the results found by the substantive coding. The purpose of these memos was to begin conceptually organizing categories relative to the core concept in support of a cohesive explanatory theory for participant concerns. As potential conceptual categories emerged, the team discussed hypothetical theoretical relationships throughout the open-coding process. Based on the reflections of the team and the memos the lead researcher selected a core category from the identified categories—”frustrated moral expectations”. The selected core category had the most explanatory power regarding the participants’ main concerns. The emergence of the core category facilitated selective coding of the transcripts for categories and properties which were related to the core category [29]. An initial draft of the theory was developed, and the entire research team worked to identify gaps in the core category, key categories, and properties. Feedback from the research team focused on the particular need to gather further data in the key categories and to develop an elegant and accessible theory illustrating the relationship between the core category, key categories, and associated properties.

## 3. Results

The selected core emergent category that explained the main participant concerns was “frustrated moral expectations”. The etymological core means: “*to deceive, disappoint, make vain [a person’s] state or condition of waiting, looking out for; desiring, hoping, longing for with anticipation [of] right behavior”.* The emerging theory comprised four categories: (1) creating unrealistic expectations; (2) trying to do the “good”; (3) minimizing and managing frustration; and (4) finding resolution. The categories included underlying psychological, social, and spiritual or existential processes that drove behaviors associated with moral suffering among participants. The identified categories (and movement between each category) were found in each of the PSP groups despite age or gender. Participants identified preconceived ideas regarding the “good” that the PSP could do (i.e., helping their community and saving lives) as a reason for entering their profession; however, participants also reported experiencing vocational events that were chronically and continually unresolved, leading to frustration, helplessness, and hopelessness. The frequent sense of hopelessness appears to culminate in problematic internal struggles. The participants’ journey through the categories did not occur linearly, as participants described their experiences across categories as dynamic throughout their careers. Thus, the categories and properties should not be seen as being on the same level and at times may juxtapose each other depending on the choices and engagement of behaviors of each participant.

### 3.1. Category 1: Creating Unrealistic Expectations

The first category is the beginning of the participants’ moral journey, starting at the beginning of their careers. The category of “creating unrealistic expectations” appears to support the basic assumption that public safety communicators, firefighters, and paramedics (and perhaps most PSP) enter their professions to help others. Participants, however, reported experiencing distress caused by being unable to help, limits in their training, and perceptions of impossible expectations from their organizations and communities. Integrated within the category of unrealistic expectations are the properties of (a) desiring the PSP moral imperative; (b) being a fixer; (c) craving acuity; and (d) requiring technical skills only.

#### 3.1.1. Desiring the PSP Moral Imperative

The property describes the Kantian “moral imperative”. Kant suggested that the categorical imperative, or universal law, to which all individuals should abide is “act only according to that maxim whereby you can, at the same time, will that it should become a universal law” (1993/1785, p. 30), suggesting that morals should be concepts a person can abide by at all times and can expect of others. Participants in the current study reported having multiple motivations for becoming PSP, but all participants described adhering to and entering the profession under a moral imperative to help people”, and by doing so, to “do good”. The formal training that the participants received, and their expectations of others (i.e., family, organizations, society), further suggested to our participants a capacity to act and fulfill the desire to “help people” in their role as a PSP; however, participants also reported being significantly compromised in their ability to help during their service, frequently experiencing that their efforts to fulfil their moral imperative were futile.

“*I think we look for a blanket statement for a blanket moral that we live by as firefighters. I would like to think we have all been hired because we are going to do the most we can to help somebody and I think that’s a blanket statement about firefighters in general*.”

(Firefighter)

“*But the existential element of what it means to be a paramedic. I am a life-saver. I am a life-preserver*.”

(Paramedic)

“*I have had some incredible calls and some not-so-incredible calls but the thread that weaves throughout every moment of my job, where I am the most fulfilled in my role and the proudest of what I do, is when my care is really advocating and helping the more marginalized people*.”

(Paramedic)

#### 3.1.2. Being a Fixer

The property of being a fixer describes taking control, a trait that becomes an all-encompassing identity for some participants (e.g., I am a helper/fixer/problem solver). In addition to being able to help, there is an expectation that PSP can take control of a situation (no matter how problematic or chaotic) and use their own decision-making, judgement, and action to resolve the associated challenges. Participants explained that PSP are purposely encouraged to be go-getters, illustrating authority, confidence, and competence to make quick decisions and take action in the face of exigent situations. The desire to help people was predicated on the notion that PSP are able to (1) enter extremely stressful, chaotic, potentially traumatic, and often surreal situations; and (2) be the person that is able to maintain their cool and accomplish what needs to be done to move the situation forward in a positive direction, restore order, and find resolution. Participants reported discovering early in their careers, however, that there are many situations in which taking control and fixing problems is impossible. Thus, feelings of helplessness, hopelessness, and failing at a professional imperative quickly replaced their sense of control.

“*Well, I personally I am a fixer, like, I solve problems or fix things. Sometimes we have scenes where we cannot fix it or solve any problems*.”

(Firefighter)

“*I think that’s exactly what makes me so proud of it is to be able to come into a situation and just kind of help manage everyone’s kind of stress and excitement and emotion and everything to just be that calming presence*.”

(Paramedic)

“*We will always get the person out. We will always do the job, but sometimes it is so catastrophic that it does not matter*.”

(Firefighter)

“*All of this work conflicts at the very core of control. Like our jobs, what we do is all based in control. Controlling scenes, mitigating, fixing, and so it’s back to that we are so uncomfortable with vulnerability and then we stigmatize vulnerability as being a bad thing or a weak thing or you are lacking something*.”

(Paramedic)

#### 3.1.3. Craving Acuity

The property describes the associations with short-term intense work leading to satisfactory resolutions. Participants described “acute-based” work as an attractive part of the PSP profession, specifically, short-term work focused in the moment and requiring concrete/specific actions with little follow-up. For some participants the acuteness was described in terms of experiencing the thrill or adrenaline associated with doing intensive calls. Paramedic participants described the acuteness as associated with being a medical detective. Participants reported discovering early in their careers that their expectations were very different from a professional reality that often involved addressing mundane, common, ridiculous, and even impossible situations.

“*Because that’s one that I see a lot of… that frustration, I see a lot of that burn out or the compassion fatigue as a lot of people want to say it but I wonder, I see it as kind of a two-pronged issue. On the one hand, you have the moral aspect to it as I am an emergency service, I am an ambulance, I should be doing ambulance-y things, but the nature of the job, if I was only ever saved for the most serious incidents, I would go out a tenth of the time*.”

(Paramedic)

“*We had a lady… I had a 911 call because she wanted her socks taken off. That’s literally why… she’s too fat to take her socks off. So I find that there’s very little training done in that and then also with the moral injury. A lot of our practitioners would just go take off the socks, be like FUCK and then just leave. And then that’s a fucking waste of resources*.”

(Paramedic)

“*And I think that’s kind of like you are saying on the low end it kind of breeds the frustration, the stress, the difficulty partly because school training you say “hey you’re a paramedic, you’re a life-saving emergency operator” but in the world you are going into there is not going to be much lifesaving*.”

(Paramedic)

#### 3.1.4. Requiring Technical Skills Only

The property describes the focus on a need for perceived competence; specifically, that PSP are highly trained in the skills needed for their specific field and to attend to life-threatening situations. Participants reported positive feelings of agency regarding helping people by using specific, applied, and task-based skills for which they have been trained. Participants also reported that, while the skills in which they were trained were useful, they were missing human skills at the onset of their career that would be needed for their occupational realities. Participants reported having to develop communication and de-escalation skills on the job and suggested such skills should have been part of specific advanced training prior to service. Participants described the absence of human skills as producing high levels of frustration early in their careers.

“*So I think the schooling itself, because it is focused so much on that task-heavy skill set, it does not value, or give time to, these more holistic approaches both in understanding you know, critical incident stress, moral injury, professional practice. That stuff seems more like a sidebar*.”

(Paramedic)

“*We do not adequately train people. It is just clinical. In EMS there is a zero focus on the social issues outside of medicine. There is a zero focus on it by anyone unless you are confronted with it and you do not know what to do*.”

(Paramedic)

“*We do lots of focus on CPR and intubation and IVs and all that kind of stuff, but the actual human side of care for the people we are serving and for ourselves is not even on the roster of curricula*.”

(Paramedic)

“*But we are put in these very dynamic decision-making situations that the clinical stuff is really easy to decide on. But it is all those other factors that are at play and we have zero training. Zero information and no systems around us to be able to reach for*.”

(Paramedic)

### 3.2. Category 2: Trying to Do the “Good”

The second category chronologically addresses issues that begin to arise while participants are working in the selected profession. Participants described that attempts to apply or do “good” lead to frustration, helplessness, and hopelessness at being unable to embody or enact their personal and professional moral imperative of helping people. Participant experiences of frustration seemed to cause moral suffering that may have led to an MI as reality eroded the moral imperative. Most PMIEs to which PSP are exposed are inherently based in the realm of ethics; specifically, the formal and informal socially or organizationally constructed expectations of behavior (Taylor, 1992). PSP exposed to PMIEs experience frustration, tension, challenge, and conflict between their deeply held desire to live out a moral imperative of helping others, and the practical realities of their environmentally contextualized vocations. As a result, PSP can be left feeling helpless, hopeless, or incapable of helping or resolving problems in a morally congruent and meaningful way. Many PSP actions may become morally futile and ethically conforming. Furthermore, as mounting unresolved moral frustrations assail their perceived integrity, their adherence to helping others as a moral imperative may cause moral suffering. Integrated within the category of trying to do the good are the properties of: (a) chronic societal problems; (b) impaired systems; and (c) organizational quagmires.

#### 3.2.1. Chronic Societal Problems

The property describes efforts to fix the unfixable and was identified by participants as a particularly challenging collection of PMIEs. Participants reported examples including, but not limited to, calls for help for: people who struggle from addictions; people with mental health problems; homelessness; elder abuse and neglect; domestic violence; and child abuse. The example PMIEs were all associated with was a sense of helplessness that did not occur for acute calls. Instead, participants described calls for help associated with chronic societal problems as being endless and irresolvable; and producing frustration, jadedness, anger, annoyance, and even hostility towards people trapped by social challenges that led to constant calls for help.

“*And so specifically to the more chronically ill, repeat calls, that type of thing where you as a paramedic are not ever seeing any change, you are just seeing the cycle of that suffering and of that harm*.”

(Paramedic)

“*I know my morals have changed when compared to when I go to an overdose call now…cause I am tired of going to these things. So for me it is like almost like when you bring them back it is like “ah fuck” cause we’re going to be back next week or two or anyways. So I think my morals have really changed, good or bad… I do not know. But I was not like that when I first started. When you first start and you bring them [addicts] back it is like “yeah, fucking right on.” And now it’s like “fuck. We’ll be back next week. See ya.” You know? It’s upsetting*.”

(Firefighter)

“*And there is like domestic violence. Where the woman goes back to… I go to the same people all the fucking time and it is like he beats the shit out of her and he is like “but we love each other.” And you just want to take him and beat the shit out of him. She has got two black eyes now and you are just like “why the fuck don’t you leave this situation?”*”

(Paramedic)

“*When kids are assaulted… when kids were mistreated by adults or whatever you are stuck dealing with the adults and reassuring them and whatever even though your focus wants to be on the kids and you do not agree with what was done to the kids*.”

(Paramedic)

#### 3.2.2. Impaired Systems

The property describes PSP acting in positions for which they felt underprepared and ill-equipped as they tried to manage challenges associated with impaired social systems not held accountable. The experience of trying to address impaired social systems sparked feelings of helplessness, worry, and annoyance that the responsibility “task” was being shifted onto PSP to fill a gap in social resources and services. Participants reported a type of moral transfer of responsibility where they were alone and personally responsible for producing a meaningful resolution to often impossible problems, while also taking responsibility for decisions, policies, or environments beyond their control. Participants reported experiencing moral frustrations regarding social and organizational system resource allocations, perceived value-conflicts, and justice. For example, public safety communicators described having to leave an elderly person lying on the floor for hours because paramedic units were already dispatched to more urgent situations the PSP deemed preventable and less ethically acceptable (e.g., overdoses, domestic disputes, and bar fights).

“*Well actually, specifically in the last 6 or 7 months, due to the [COVID-19] pandemic, we had to do some changes into our response plans to help handle the extra volume of the pandemic, we are actually starting to hold calls that we normally never used to hold at all. So when we have a low priority event, normally in the past, we would just deploy and now we are holding back resources and only responding to those low-priority events when we have an adequate amount of resources available*.”

(Dispatcher)

“*You go into somebody’s drug house and you see a drug addict that is, you know, laying on the floor unconscious and there are two or three kids running around and there is drugs all over the place and you are you know..thinking “jeez, something needs to be done here.” Like as a firefighter I have zero capability to take those kids away you know, um, we can phone the police and let them know but that is it. You know, you do not know whatever happens after that*.”

(Firefighter)

“*I think a lot of it is caused by our culture. It is caused by the system in which we operate and part of it has to do, like you said, this is never what we were intended to do. Paramedics should not be mental health responders but we do not have mental health responders, we have paramedics*.”

(Paramedic)

“*Leaving an old lady laying on the floor for 60 min with a broken hip and you feel bad about that..: Because we deal with that a lot when you have to make your decisions about where you send your resources and um, there could be someone who is in a tremendous amount of pain on the floor in their apartment but they’re not a priority? So it is just something that we deal with that is like “well is that the right thing to do? Is that morally just?”*”

(Call taker/Dispatcher)

#### 3.2.3. Organizational Quagmires

The property describes things that should be fixable but are left unresolved serving as primary sources of PMIEs. In line with related research on occupational stressors impacting mental health [24,25], participants reported organizational decisions and perceived level of support as contributing to or amplifying exposure to avoidable PMIEs and associated moral suffering. Many of the organizational PMIEs to which PSP were exposed revolved around ethical conflicts within the workplace, toxic cultures, sexism, micro-aggressions facilitated by paramilitary and hierarchical structures, insufficient resources, and colleagues and leadership suffering with mental health challenges, including MI. Many participants reported feeling their employers viewed them as machines to do a job, rather than as human beings. Participants reported being criticized and witnessing coworkers being criticized or punished for sharing mental health challenges caused by the emotional toll of serving as PSP. In contrast, participants also reported that positive changes to culture from leadership made significant differences, as they felt understood, heard, and supported by their organizations.

“*It is not normally the gruesome things you see or anything like that. It is usually the calls you have been questioned on what you have done and if something has gone wrong and just not having that support or even having anybody to talk to right away*.”

(Paramedic)

“*We are sitting here telling you the chronic injury is from our workplace culture and our lack of support. That is what is continually harming*.”

(Paramedic)

“*So, when it comes down to… well people always say “well just take a time out. Take a time out.” Um, the work does not stop. So our needs whereas other people say “well just ground the ambulance. Take them out of service.” Okay that works for them that does not really work in our Centre*”

(Dispatcher)

“*Getting back to the question, what I really want them to know is what do you need to humanize your frontline workers? You need to look at them more than just meat in a seat*.”

(Paramedic)

“*Leadership here in XXX right now is probably the best we have ever seen and right now would be the perfect time to, I do not know… Implement something because these guys understand. They have been on the floor, they understand how things work and how things are running. Right now would be the perfect time and those guys get it*.”

(Firefighter)

### 3.3. Category 3: Minimizing and Managing the Moral Frustration

The third category stems directly from the second category (participants often loop between these two categories) as participants attempt to find processes or ways of coping in efforts to manage and minimize their moral frustrations while still working in their selected occupation. PSP reported repeated PMIE exposures and then being left alone to manage the emotional, psychological, and spiritual sequelae resulting in effectively two pathways: (a) disintegrative paths using the processes of working to shove it down and moral compromising, and (b) integrative path using the process of gaining soft skills. For the disintegrative path, participants reported that unresolved exposures accumulate over time, causing progressive mental health challenges akin to burnout [26]; for example, participants reported that repeated PMIEs could lead to boredom, annoyance, indifference, anger, despair, and apathy. Conversely, some participants recognized their moral suffering and sought to address it independently though gaining soft skills (e.g., emotional intelligence, counselling, grief work, etc.). The selection of these two pathways (processes) by participants appeared to be influenced by organizational culture, social and familial factors, and personal risk and resiliency factors. Integrated within the category of minimizing and managing moral frustration are properties of (a) working to shove it down; (b) moral compromising; and (c) gaining soft skills.

#### 3.3.1. Working to Shove It Down

The property describes an early coping strategy for managing moral suffering. Participants reported using suppression and avoidance as strategies deemed acceptable and required by their organizations for coping with exposure to PMIEs or potentially psychologically traumatic events [6]. For example, participants described explicit organizational and peer expectations that PSP should be inherently able to cope with experiences considered part of the job (e.g., you signed up for this, this is what it means to be a PSP). Use of avoidance as a coping strategy was associated with fear of reprisals for being honest or vulnerable about mental health, as well as stigma driven by feelings of shame, humiliation, rejection, insecurity, and inadequacy.

“*And some of them, and I have been told this to my face. “Look, if these people are too weak to work here, I don’t want them here.” So that attitude is still there. So a lot of people get… and it happens here in XXX, where paramedics are getting pushed out or fire fighters are getting pushed out because the employer does not want them back because they feel they are too weak*.”

(Paramedic)

“*Yeah, it is not a job that I never endorse people to suggest it as a career. I do not believe it to be a career, especially at a working position. I do not believe a person can do this for 20, 30 years and come out healthy*.”

(Dispatcher)

“*Oh no I have to keep working and then that is fine because a month from now I am quitting because I am done with this stupid job and nobody takes care of me and so on and so forth and you just become another one of those burn out statistics and then everybody is sitting there wondering “why do paramedics burn out so much? Maybe we’re not whipping them hard enough. Let’s whip them harder.”*”

(Paramedic)

“*Nobody talked about it* [trauma and MI]. *So you just kind of sat and stewed on it and if you talked about it you were a wimp. But yeah, the older EMTs or whatever you want to call them, they are jaded, a little salty because we have had years where you just put it down, shove it down, shove it down,… like months or years or whatever, this built up, and yeah, you snap or you burn out or….*“

(Paramedic)

#### 3.3.2. Moral Compromising

The property describes the most common coping strategy for managing participants used to manage moral frustration and moral suffering. Participants reported experiencing temporary relief from moral frustration and moral suffering by creating cognitive and emotional space between themselves and their original moral imperative. For example, participants reported increasingly accepting cognitions such as, “*the world is shit”; “people are horrible”; “it does not matter what I do”; “my work is pointless or futile”* in attempts to realign their expectations. Participants associated moral compromising with annoyance, disappointment, skepticism, dismissiveness, numbness, anger, being judgmental, bitterness, and aggressiveness. Participants also associated moral compromising with changes in behaviors and justifying behaviors that would have previously been incongruent with their personal values.

“*You get sick of going to some of these overdoses but then, like I have had buddies who have struggled with addictions before and you know, one guy he ended up dying from it. You know? So was my buddy, you know, a shit bag? Yeah. By my definition. You know, he was. But he was still my buddy. Someone that I grew up with. Went to high school with, you know? So it is weird how you can have two different people and still label them the same but one was your friend and one you could really care less about*.”

(Firefighter)

“*So I think there are a lot of moral assumptions or implications we make about our patients and they tend to be far less than flattering and having seen the way some paramedics are treating patients like this, I can say if they treated my family member like that, I would be going straight to the College and saying “take their license*”.”

(Paramedic)

“*Mine have changed over time. So I found that my morals have kind of adjusted into realistic expectancy and that has gone from we need to save everyone to what good are we going to do certain patient populations*.”

(Paramedic)

“*I woul;d say moral injury yeah because I did not know this stuff until I had gotten in.. we were talking about just going we will dehumanize people on the way to calls because you need to be able to do that in order to survive…So like the humanity aspect of it and you are like fuck there is nothing I can do about it though. Like I can treat them with respect as an individual. I can be cognizant of my own cognitive biases that form and trying to advocate for them and you try to rehumanize after and you are like that is really sad…and it is really fucking sad and you are like society has failed him… but you can not spend too much time on it because you will just burn out*.”

(Paramedic)

“*When I use the term jaded it could be how they personally feel and they have gotten to the point where they are burnt out.. Our frequent fliers… You know you are picking them up and it is the same thing over and over again and you are seeing them make the same bad life choices and it is frustrating so I think that the term jaded is definitely. The exposure has just made them to the point that they no longer, I guess the way to say, they no longer feel empathy or sympathy, but they have almost burned all of that out*.”

(Paramedic)

#### 3.3.3. Gaining Soft Skills

The property describes a less often reported coping strategy that may be associated with increased service experience. For some participants this coping strategy included going to therapy; learning more soft skills, such as improving their personal communication; learning about concepts, such as compassion fatigue, burnout, secondary traumatic stress, and MI; learning about their own grieving processes and preferences and allowing space to grieve; and finding spiritually meaningful and fulfilling practices. Irrespective of the specific activity, participants reported wanting to learn how to understand what was happening to them; accept their reactions; and integrate their frustrations in meaningful ways. Gaining soft skills appeared to contrast moral compromise because participants actively engaged with their experiences and sought to contradict notions of helplessness, hopelessness, and despair.

“*I think understanding morals and your values though as well can help you sleep cause we talk about if you are weak or this and that and you do not want to get help and you do not want to show weakness. But if I go to a call and there are eight of us on a call and seven of the guys are completely fine with it, why am I not fine with it? If I do not understand my morals and my values, maybe that is the difference*.”

(Firefighter)

“*It is just our men are hurting and we need to stop. We need to make it okay to be human. Just to be messy. We are messy human beings and we are showing up and caring for messy human beings*.”

(Paramedic)

“*You can take this but our schooling is so brief and so limited that we talk about “hey remember to be compassionate with your patients. Remember to be empathetic of their situations.” Okay, but how? How do I walk into this situation where on the one hand a drunk driver has killed an entire family and he is the only one alive and how am I going to now take care of him. Or on the other hand how do I stay respectful to this intoxicated, clearly homeless, has not washed in a very long time and smells terrible, how do I treat that person with the same care and respect as the 65-year-old grandmother I go to later who fell and broke her hip*.”

(Paramedic)

“*The day to day is those soft skills. The day to day is the ability to communicate with the patient and to approach it with that open minded neutrality of “hey you called 911, what’s up?” Not “oh Jesus Christ, it’s you again, get in the ambulance.”*”

(Paramedic)

### 3.4. Category 4: Finding Resolution

The fourth category describes resolution to moral suffering and appears focused on the outcomes associated with coping solutions. Participants often engaged in different categories of coping (especially “trying to do the good ‘‘and “minimizing and managing moral frustrations”), but reported that, ultimately, there were only two resolutions to MI: (a) escaping; or (b) ontological transformation.

#### 3.4.1. Escaping

The property describes a coping solution deemed by most participants as ubiquitously necessary, at least in the short term. The specific escape behaviors and intensities varied from moving into a new department, role, unit, or geographic location, to leaving the profession, to dying by suicide. A primary driver of escape behavior identified by participants was the desire to remove themselves from the sources of their moral frustration and moral suffering, specifically, people (society), and their PSP organizations.

“*…In my workplace each time we attend a funeral for one of our colleagues [who died by suicide], our conversations after are like “our men are dying.” It's our men who are dying. And we can point to some tangible reasons why and we get the “here is your EAP number, call if you need help“*.”

(Paramedic)

“*And more harm actually because… and yeah actually more people probably exiting the profession because they will be a little bit more woke and be like “holy fuck, I need to get out of this shit before it kills me.”*”

(Paramedic)

“*Sorry the work environment right now, I know in our organization, is very, very crappy. People are leaving. We have lost 12 medics in the last year. Never mind EMTs on top of that. We can recruit anybody, we can not keep them. Just the whole, the whole lack of support from management*.”

(Paramedic)

“*Yeah, I think for me personally though, had I not transitioned into a promoted position, I am not sure how much longer I would have been here. Would have stayed here… I felt I was starting to lose my edge and losing… I was not as good at the job as I was previous to that. So I felt like I was starting to lose something else… Something deeper*.”

(Dispatcher)

#### 3.4.2. Ontological Transformation

The property describes a coping solution referenced by a few participants, specifically, that remaining a PSP depended on restructuring their basic assumptions on, and expectations of, their profession. Participants reported the ontological transformation (i.e., changing their whole being) as being predicated on revising their unrealistic expectations (category 1) such that the subsequent moral frustrations became less frequent, thereby minimizing the associated moral suffering. Participants reported needing to understand and accept their realistic capacity to change the impaired social systems (category 2), to acquire soft skills (category 3), and to create vocational meaning and purpose through small acts of good (category 1), despite the pervasive and perpetual challenges they face. Participants also underscored the importance of maintaining relationships and a life outside of their PSP work to maintain their own independent identity. Realistic expectation management and self-compassion appear to help mitigate the massive weight of repeated exposures to PMIEs and potentially psychologically traumatic events, while still allowing participants to fulfill their moral imperative and serve as PSP.

“*From a user perspective you know, almost like and it really should be ongoing, almost a self-checklist of key emotions that some people might be feeling that might be triggers that maybe they are having some issues that need to be treated. Yeah, some kind of a self-check that they would do periodically would be important for them*.”

(Dispatcher)

“*Like a paramedic is who I am, that is in my blood. I breathe it. And my resilience has come from having a personal identity that is different from my profession. It is not hinged on my profession… that does not mean that we do not experience suffering throughout our job and from our work but it is different from internalizing the badness that goes on*.”

(Paramedic)

“*And what got me out of it was just adjusting the mindset and saying, “hey I’m a paramedic but that doesn’t mean that’s all I am.” I am somebody that helps the patients and somebody that can root in and start asking questions and am somebody who understands the health care system superficially enough that I can guide people through it. And just kind of enriching my role and also the patient situation and realizing that there is more to paramedicine than just picking someone up and taking them to the hospital which is unfortunately what we focus on in school*.”

(Paramedic)

“*If we want paramedics to actually stand the test of time, we need to give them the tools to make it through and not just the tools to make it through because a lot of paramedics are making it through now as burnt out, jaded, terrible people but how do we make them…a little shiny? Or keep the edges from getting too sharp*?”

(Paramedic)

## 4. Discussion

The current research examined whether MI as a concept is relevant to the experiences of frontline public safety communicators, firefighters, and paramedics. Results indicate that MI was highly relevant to these populations. Central to PSP struggles with MI is the suffering associated with having moral desires and expectations repeatedly go unfulfilled. The potentially psychologically traumatic events experienced by PSP seem to focus on *what happened*. The PMIEs to which PSP are exposed seem to relate to *what should have happened* [30]. While limited differences in PMIEs were found between PSP professional groups, specific roles and responsibilities seemed to influence perceptions of events as potentially morally injurious. For example, the different PSP professional roles for calls involving drug use appeared to impact how each PSP interacted with the people involved. Firefighters treated the drug overdose, while paramedics additionally attempted to help the patient by advocating and providing additional health resources. If PMIEs differ between PSP groups as a function of professional responsibilities, additional research would help to better delineate MI and clarify pathways for tailored solutions. These results also highlight that MI may be a relevant construct in other PSP populations, e.g., frontline services personnel or other trauma-affected populations.

PSP organizations are typically paramilitary, and PSP have often been characterized as experiencing potentially psychologically traumatic events similar to military personnel (i.e., violence, gruesome scenes, risk of death) [31]. The current results, however, support previous indications of important differences [32]. For public safety communicators, paramedics, and firefighters, MI was conceptualized as driven by exposure to PMIEs that frustrated moral expectations regarding chronic and unresolved societal and organizational realities that lead to protracted moral suffering and mental health challenges. Differences in PMIE for PSP and military may be because of the realm (i.e., moral versus ethical) in which PMIEs occur. The moral realm relates to the fundamental rules of right and wrong which hold us together as a society (i.e., killing is wrong), a common PMIE for military personnel [33]. Conversely, PMIEs based in the ethical realm involve the contextualization or application of morals and were frequently experienced by participants. For PSP, PMIEs appeared associated with inabilities to do what they thought was “right”, rather than directly breaking fundamental societal rules which they knew were “wrong”.

Another notable difference in PMIEs between military personnel and PSP is managing chronic exposure to systemic societal problems that are not easily addressed or rectified, including impaired social and healthcare systems. Participants experienced moral suffering as a result of frequent encounters with people seeking or requiring help that PSP could not provide, and for some this likely evolved into MI. Responding to the same people or situations time and again in matters that went beyond the PSP scope of practice highlighted gaps in social and health services for which PSP became the failsafe. Over time, participants were negatively affected by having to fulfill this role. Being placed in untenable situations may also explain why PSP described MI as involving feelings of resentment, bitterness, anger, disappointment, disillusionment, sadness, despair, hurt, frustration, helplessness, and hopelessness, which differs from military personnel who described MI as involving feelings of guilt and shame [34]. The emotions described by PSP appear to reflect transgressions based on others or on betrayal, along with feelings associated with forced helplessness. Accordingly, PSP may identify more PMIEs driven by the actions or inactions of others, or of organizations, rather than PMIEs driven by their own behaviors [35].

Understanding the role of both moral and ethical emotions [30] may support a more comprehensive understanding about moral suffering and MI. For example, PMIE resolutions may differ depending on whether the resulting emotions are self-based (e.g., guilt, shame, condemnation), other-based (e.g., anger, bitterness, resentment), or betrayal-based (e.g., disappointment, sadness, helplessness). If so, then more nuanced assessment tools and treatment approaches would be required to tailor and provide effective solutions. The nuance and tailoring may be extremely important for PSP who can reasonably expect careers involving exposures to multiple PMIEs and kinds of PMIEs, in addition to a diversity of potentially psychologically traumatic events, all of which may facilitate mental health challenges. For example, chronic exposure to situations where mastery and sense of control are impeded may have an effect on PSP self-efficacy, an important factor in reducing stress and improving wellness [36,37,38,39]. Additionally, this impeded mastery may have an impact on perceptions of moral agency which is an important component of moral resilience and identity [40,41]. Filling gaps in the social system with more appropriate resources could decrease the moral struggle and psychological stress that PSP encounter daily.

The current study results indicate that two broad pathways may be used to address moral suffering after PMIE exposure: disintegration or integration [42]. Participants identified two main disintegration practices—shoving it down and moral compromising. The practices may parallel the coping strategies described for persons experiencing PTSD. The practice of “shoving it down” may parallel avoidance [43,44] or numbing [43,44,45,46] experiences that can follow potentially psychologically traumatic events and serve as problematic coping strategies [18]. The practice of moral compromising may be used to manage the impact of exposure to PMIEs wherein holding onto a “good” would require too great a personal sacrifice [46]. However, such moral compromising, may lead to mental health challenges and problematic decision-making due to of violations to personal moral integrity [47] that compromise the self [48]. MI appears to involve fractures to personal identity at the level of “self” [49,50], resulting in a slow disintegration of selfhood, identity, and the ability to engage in the world (i.e., relationality). Other researchers have evidence that PSP report feeling alienated from their past selves as a result of service [51], which may be explained in part by pervasive MI resulting from an inability to live congruently with oneself [52].

The final category of “finding resolution” highlights the challenges of unresolved moral suffering leading to MI, resignations, and suicide [53]. Unresolved moral frustration, moral suffering, compassion fatigue, and burnout were proffered by participants as potential reasons PSP leave their professions, a pattern also evidenced among healthcare workers [47,48]. Research on suicide and MI has predominantly focused on military personnel [49] and veterans [50,51,52]. Our results provide preliminary evidence of similar associations between MI, resignations, and suicide among PSP. Given the significant and severe negative occupational and health outcomes associated with MI, PSP appear to require a broader approach to managing MI and other PTSIs that includes increasing the voice of employees in the workplace [53], as well as improving organizational and employee wellness [54]. Antecedents to organizational support and psychological safety include selecting trustworthy, empathetic, supportive, and moral leaders with positive leadership styles [55]. Even more broadly, leaders and policy makers are urged to consider the balance of the social and economic influences of health within their area of responsibility and how PSP are a part of both public safety and public health.

In addition to organizational changes, PSP may benefit from learning specific skills to navigate exposure to PMIEs and mitigate moral suffering. In parallel with the existing research, participants indicated that they were able to ease their moral suffering by: increasing their emotional intelligence; engaging in mental health education; acknowledging their grief [55]; re-finding meaning and purpose [56]; engaging in compassion and forgiveness practices [57,58]; identifying personal values [59,60]; and seeking social support [61]. The importance of these integrative practices may be that participants did not seek to “solve” or mitigate the external moral stressors or suffering, but to build an internal capacity and competence to accept, manage, and rectify moral suffering. Through this, our participants illustrated that moral resilience is possible despite chronic exposure to PMIEs [41]. However, moral resilience, should not be interpreted as an inoculation to moral suffering, but an increased tolerance to PMIEs exposures. PSP may benefit from learning and being supported in the regular practice of specific soft skills to help navigate PMIE exposures. This may mitigate MI, address moral suffering, and help to improve PSP mental health by addressing other mechanisms of injury beyond exposures to potentially psychologically traumatic events. A similar approach to work-place psychological injuries should also be explored for all frontline services personnel, especially in light of the COVID-19 pandemic where exposures to PMIEs is believed to have substantially increased [62]. Applicability of MI may therefore expand beyond traditionally deemed trauma-affected populations.

### Limitations

The current study has several theoretical and practical limitations. Theoretically, the proffered theory was derived from the experiences of Canadian public safety communicators, firefighters, and paramedics, which makes generalizability to all PSP unlikely. The broad inclusion criteria meant participants represented a spectrum of experiences, and years of service (e.g., most participants had served between 10–15 years as a PSP) further compounds the ability to theoretically identify MI in each of the specific PSP populations. Interestingly, potential differences based on their diversity were not found in the current data; however, it is unclear if these differences would be found in a larger sample size.

Practical limitations also include the following. First, participants were not being treated for MI or PTSI; therefore, the data gathered may have been skewed towards participants who were seeking some form of professional mental health treatment or who were already knowledgeable on the topic. Second, MI is not a prolifically recognized construct among PSP, as such, PSP may not have volunteered because we did not clarify the potential relevance. Third, participants were recruited using convenience sampling, so there may be unidentified problematic selection biases. Fourth, this study occurred during the COVID-19 pandemic which may have impacted the ability to recruit participants. This timing also resulted in the data collection occurring remotely and online. The contextual reality may also have impacted the experiences of participants and skewed the data regarding PMIEs and MI. Future research should explore MI in light of different levels of PSP occupation (i.e., frontline versus middle management, versus senior management) and with a focus on organizational challenges related to MI. Additionally, continued exploration of MI should consider diversity, equity, and inclusion principles.

## 5. Conclusions

The current study was designed to examine whether PSP perceive MI as relevant. The current results evidence that MI appears to be relevant based on our participants strongly identifying the harms caused by chronic exposures to PMIEs. The large variety of PSP environments, roles, and PMIEs, underscore the potential benefits of better understanding the associated risks for PSP mental health.

## Data Availability

Data is only available on request due to privacy/ethical restrictions. The data that supports the findings of this study are available on request from the corresponding author, L.S.M. The data are not publicly available due to their containing information that could compromise the privacy of research participants.
